# Patient Experience and Satisfaction with Inpatient Service: Development of Short Form Survey Instrument Measuring the Core Aspect of Inpatient Experience

**DOI:** 10.1371/journal.pone.0122299

**Published:** 2015-04-10

**Authors:** Eliza L. Y. Wong, Angela Coulter, Paul Hewitson, Annie W. L. Cheung, Carrie H. K. Yam, Siu fai Lui, Wilson W. S. Tam, Eng-kiong Yeoh

**Affiliations:** 1 The Jockey Club School of Public Health and Primary Care, Faculty of Medicine, The Chinese University of Hong Kong, Hong Kong, China; 2 Nuffield Department of Population Health, University of Oxford, Oxford, United Kingdom; University of California San Diego, UNITED STATES

## Abstract

Patient experience reflects quality of care from the patients’ perspective; therefore, patients’ experiences are important data in the evaluation of the quality of health services. The development of an abbreviated, reliable and valid instrument for measuring inpatients’ experience would reflect the key aspect of inpatient care from patients’ perspective as well as facilitate quality improvement by cultivating patient engagement and allow the trends in patient satisfaction and experience to be measured regularly. The study developed a short-form inpatient instrument and tested its ability to capture a core set of inpatients’ experiences. The Hong Kong Inpatient Experience Questionnaire (HKIEQ) was established in 2010; it is an adaptation of the General Inpatient Questionnaire of the Care Quality Commission created by the Picker Institute in United Kingdom. This study used a consensus conference and a cross-sectional validation survey to create and validate a short-form of the Hong Kong Inpatient Experience Questionnaire (SF-HKIEQ). The short-form, the SF-HKIEQ, consisted of 18 items derived from the HKIEQ. The 18 items mainly covered relational aspects of care under four dimensions of the patient’s journey: hospital staff, patient care and treatment, information on leaving the hospital, and overall impression. The SF-HKIEQ had a high degree of face validity, construct validity and internal reliability. The validated SF-HKIEQ reflects the relevant core aspects of inpatients’ experience in a hospital setting. It provides a quick reference tool for quality improvement purposes and a platform that allows both healthcare staff and patients to monitor the quality of hospital care over time.

## Background

Input from patients is a fundamental feature of patient-centred care [1,2]. Direct feedback from patients is considered the best way to measure the quality of their experiences [3,4]. It has proved useful to ask patients to report on detailed aspects of what happened during a specific care episode, rather than asking them to rate their satisfaction using general evaluation categories [4–6]. Standardized patient experience questionnaires are widely used for assessing the quality of healthcare from the patient’s perspective [7]. One of the most widely used patient experience surveys is the Adult Inpatient Survey, which was originally developed for use in England by the Picker Institute Europe [8–10]. A validated core set of items (PPE-15) that identified key aspects of patients’ experience was subsequently developed in 2002 [11,12].

Patients’ experience is influenced by the structure of the health system. Hong Kong is an ex-British colony and therefore has a tax-base funded health system and hospital settings similar to those in the United Kingdom. In 2010, a validated instrument measuring patients’ experience, the Hong Kong Inpatient Experience Questionnaire (HKIEQ), was developed by adapting the General Inpatient Questionnaire of the Care Quality Commission in England and integrating it with findings from a local validation study [13,14]. The HKIEQ has good structural validity and reflects the multidimensionality of patients’ experiences of different aspects of care during hospitalization. The questionnaire consists of 54 items, and the responses can be turned into scores relating to specific, actionable quality improvement measures. However, the length of the questionnaire limits its use. The development of a short-form version of HKIEQ is thus important for maintaining the momentum of the quality improvement culture and for soliciting patients’ views on a regular basis. This study designed a Short-form Hong Kong Inpatient Experience Questionnaire (SF-HKIEQ) consisting of a core set of items from the HKIEQ that reflect the most important components of patient experiences from the perspectives of both patients and healthcare professionals.

## Methods

### Study design

The HKIEQ consists of 54 evaluative items in 9 dimensions [13,14]. To develop a short-form version, a core set of items was identified using a two-stage approach: (1) a consensus conference; and (2) a cross-sectional validation survey.

#### Stage 1: Consensus Conference

A consensus conference was conducted to obtain experts’ opinions on the criteria for selecting a core set of items from the HKIEQ, and further, to develop a preliminary version of the short-form for testing in a validation survey in the second stage. Delphi methodology—a systematic approach that engages a group of experts in a process to derive consensus by rating a research framework—was adopted [15,16]. The characteristics of the Delphi method include anonymity; iteration; controlled feedback; and statistical group response, which allow participants to provide their initial response and then made subsequent changes after viewing the group’s responses [17,18]. Based on the findings of the 2010 Patient Satisfaction Survey [19], the experts recommended the following psychometric criteria [11,12,20,21]:

1. an item included in the core set should be applicable to as many respondents as possible;
2. core set measures should be highly correlated with the original measures at 0.9 or above [22];
3. the internal consistency of the core set should reach 0.7 or above (KR-20’ is effectively Cronbach’s alpha statistic for dichotomous variables) [23,24]; and
4. item-to-total correlations, corrected for overlap, should exceed 0.3 for items within a measure [25].

The literature suggests that a sample size of 15 should be sufficient to yield saturation on the face-validity of a questionnaire [16]. In Hong Kong, all of the public hospitals are managed by the Hospital Authority under seven geographical clusters. We therefore invited 2–3 health professionals currently working on healthcare quality, patient engagement or patient satisfaction surveys from each of the seven geographical clusters to attend the consensus conference.

#### Stage 2: Validation Survey

We carried out a cross-sectional telephone survey of the preliminary scale and evaluated its acceptability, appropriateness and psychometric properties. The target population was Hong Kong citizens with a Hong Kong Identity Card, 18 years or older, Cantonese-speaking, who had had at least one overnight stay in one of the medical departments (surgery, orthopaedics and traumatology, emergency medicine, gynaecology, ear/nose/throat, clinical oncology and ophthalmology) at one of the 25 major acute and rehabilitation public hospitals in Hong Kong. Users of psychiatric or obstetric care, dentistry, hospice, infirmary, paediatrics, intensive care, anaesthesiology, and “other” departments were excluded from the survey. We estimated that about 500 participants would be sufficient for validation purposes, taking into consideration the number of items [26–29].

### Data Collection

First, a summary of suggested criteria for developing a core set of items was presented to participants during the first round of the consensus conference. The experts were invited to express their views and then indicate their preferences for the proposed approaches. In the second round of the consensus conference, an amended version of the core set based on the chosen criteria was generated and the experts were asked to re-rate their preferences. Delphi methodology suggests that a minimum 75% level of consensus should be achieved; the number of rating rounds to achieve consensus depends on the level of consensus reached in each round [17].

Once the framework and face validity of the core set were established, a cross-sectional telephone survey was conducted. The participants were 500 patients who had been discharged from one of the 25 major acute and rehabilitation Hong Kong public hospital 48 hours to 1 month before the interviews, which were conducted between November 2012 and December 2012. The participants were asked to report their most recent inpatient experience using both the long-form HKIEQ and the short-form version [14].

### Statistical Analysis

At the consensus conference, the choice of development approach was recorded using descriptive statistics to assess when the pre-specified level of 75% agreement was reached. Qualitative feedback about the criteria revisions was also recorded. For the validation survey, the data entry, management and analysis were performed using PASW version 18.0. Double-entry data input was used to ensure accuracy. The descriptive statistics of the sampled demographics were presented using frequencies and percentages or mean values, as appropriate. The effects of issues such as the percentage of missing values on individual items and the total completion interview time on the acceptability of the questionnaire were assessed [24,30]. Missing values were estimated based on the proportion of patients who refused to answer items or answered “Don’t know”/“Forgot”. The average interview completion time was calculated to test the appropriateness of the survey instrument length.

Each item on both the HKIEQ and the short-form was coded as a dichotomous “problem score” that indicates the presence or absence of a problem [11]. A problem was defined as a subjectively evaluated aspect of health care that could be improved. For example, the categorical answers for the item Did you have confidence and trust in the doctors treating you? was: (1) Yes, always; (2) Yes, sometimes; and (3) No. Options (2) and (3) for this items were coded as a “problem” for this aspect of care. To evaluate the construct validity of the short-form, a Spearman rank correlation of the summative problem scores was computed to compare the short-form and the HKIEQ. It was suggested that the selected core items should be highly correlated with the original measures, with a coefficient of 0.9 or above [22]. The Cronbach’s alpha coefficients (KR-20) and the item-to-total coefficient (corrected for overlap) were then estimated to test the internal consistency reliability of the instrument, which assessed whether the items in the questionnaire measured the same concept. A KR-20 coefficient of at least 0.7 generally indicates good reliability among scales, whereas a coefficient below 0.6 suggests the item should be rechecked [23,24]. The Spearman correlation coefficient (ρ) should exceed 0.3 for items within a measure [25].

### Ethical Consideration

Ethical approval was obtained from the Clinical Research Ethics Committees of the Hospital Authority. All of the participants were informed of their rights, and were given information about the purpose of the study and details of the research procedures before the telephone interview. Verbal informed consent was obtained from each of the participants before the interview started. The participants were allowed to withdraw from the study at any point. Initial screening for eligible patients was conducted and their agreement to participate in the study was obtained by hospital staff and verified by our research team. All of the participants’ consents or refusals were documented by the interviewer. To ensure the quality of the interviews, the interviewers were monitored. All of the data were kept confidential and anonymous.

## Results

**Fig 1:**
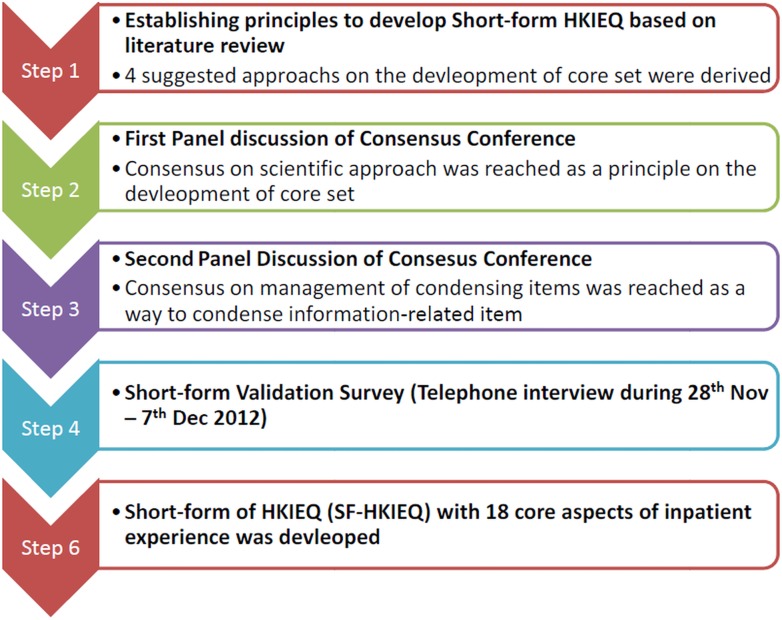
Workflow of development of core patient experience items.

The study workflow is shown in Fig 1.

### Stage 1: Consensus Conference

Fifteen nominated experts from public hospitals were invited to attend the consensus conference. Three refused due to unavailability and 12 experts attended. The experts had a mean of 5.8 years (SD: 3.2 years) of working experience in the field of patient engagement. The target minimum of 75% consensus on the development of the preliminary version of the core set of HKIEQ items was reached with two rounds of consensus conference.

#### First Round of Consensus Conference

In the first round of the consensus conference, three approaches to the development of a core set of items were presented: (1) a psychometric approach using the four psychometric criteria listed above; (2) a problem-oriented approach focusing on the items that achieved low scores in the first population-based Patient Satisfaction Survey in 2010; and (3) a domain-oriented approach that included at least one item from each of the nine domains of the HKIEQ selected by ranking the item-to-total correlations. All of the experts (12/12, 100%) agreed to adopt the psychometric approach; 22 items were thereafter selected for inclusion as a core set (Table 1). In addition, the participants suggested that items covering similar topics needed to be rephrased to make the instrument simple and brief.

**Table 1:** Selected core items from Hong Kong Inpatient Experience Questionnaire.

Patient Journey	Spearman Correlation	Selected for Core
**Admission to Hospital (9 items)**		(0 item)
Information about condition/treatment in the A&E department (for A&E admission only)	NA	
Length of time before being examined by a doctor (for A&E admission only)	NA	
Length of time before being admitted to a bed on a ward (for A&E admission only)	NA	
Length of time before being admitted to a bed on a ward (for plan admission only)	NA	
Length of time before being admitted to a bed on a ward (for other admission only)	NA	
Perception of length of time to get to a bed on a ward	0.234	
Length of time on the waiting list before admission (for plan admission only)	NA	
Any choice of admission dates (for plan admission only)	NA	
Changing admission date by hospital (for plan admission only)	NA	
**Staying at Hospital and Ward—Environment, Food and Facilities (9 items)**		(0 item)
Bothering by noise at night from other patients	0.150	
Bothering by noise at night from hospital staff	0.123	
Cleanness of hospital room/ward	0.192	
Cleanness of toilets/bathrooms in hospital	0.214	
Anywhere for keeping personal belongings	0.046	
Seeing any posters/ leaflets asking patients/visitors to wash hands	0.228	
Providing hand-wash fluid/ gels for patients and visitors	0.165	
Rating of hospital food	0.224	
Any choice of food	0.213	
**Staying at Hospital and Ward—Hospital Staff (5 items)**		(4 items)
Receiving a clear answer of the questions from doctor	0.484	Selected
Having confidence/trust in doctors	0.378	Selected
Receiving a clear answer of the questions from nurse	0.354	Selected
Having confidence/ trust in nurses	0.350	Selected
Enough nurses on duty to care for patients	0.207	
**Staying at Hospital and Ward—Patient Care and Treatments (12 items)**		(8 items)
Say something quite different among staff	0.111	
Involving in decisions about care/treatment/procedure	0.266	
Receiving any information about condition, treatment or procedure	0.387	Selected
Having enough opportunity to talk to doctor by family member	0.584	Selected
Discussing / comforting patient’s worries/fears by healthcare staff	0.607	Selected
Any privacy when discussing condition/ treatment/procedure	0.349	Selected
Any privacy when being examined or treated	0.261	
Doing everything to control pain by hospital staff	0.443	Selected
Length of time usually take for answering button by hospital staff	0.220	
Getting enough help needed from hospital staff	0.314	Selected
Receiving details of treatment/ operation/ procedure results beforehand	0.453	Selected
Receiving details of treatment/ operation/ procedure results afterward	0.483	Selected
**Leaving Hospital (10 items)**		(5 items)
Involving in decisions about discharge	0.205	
Length of time of the discharge delay	0.145	
Clearly explaining the purpose of your medicine by hospital staff	0.299	
Telling about medication side effects by hospital staff	0.421	Selected
Telling how to take your medication	0.304	Selected
Giving clear information about medicines	0.239	
Telling danger signals watch for after discharge	0.552	Selected
Giving family all information they needed for patients’ care and recovery	0.673	Selected
Telling who to contact after left hospital by hospital staff	0.487	Selected
Feeling the given contact information useful	0.205	
**Overall Impression (9 items)**		(5 items)
Being treated with respect and dignity in hospital	0.387	Selected
Rating of the care received from doctors	0.451	Selected
Rating of the care received from nurses	0.412	Selected
Rating of the care received from healthcare assistants	0.363	Selected
Rate of the overall care received	0.449	Selected
Being asked to give views on quality of care	0.046	
Any drop box for your opinions/ complaints related to hospital	0.169	
Expressing any opinions about the recent care received in hospital	0.215	
Complaining about the recent care received in hospital	0.211	
**Total Number of Items from HKIEQ selected as Core Items**		**22 items**

NA: Excluded for Spearman correlation analysis because they do not applicable to all patients

#### Second Round of Consensus Conference

In the second round of the consensus conference, two ways of condensing items were discussed (Table 2): condensing the five items related to information seeking into two items; and condensing the seven items related to specific job roles (physician, nurse and healthcare assistant) into three items. Four models based on the two ways of condensing the items were presented: (1) condensing for both information and role-related items; (2) condensing for information questions only, (3) condensing for role-related items only, and (4) no item condensing. One hundred per cent of the participants agreed that model 2, condensing for information only, was the best strategy. On this basis, a preliminary version of the short-form consisting of 19 items was developed.

**Table 2:** Two suggested ways to condense items.

**(1) Condense for Information-related Items**	
Condense 1.1	(i) Was there enough information about your condition, treatment or procedure given to you?
	(ii) Beforehand, were you told the detail aspects of the treatment, operation or procedure and its results in a way you could understand?
	(iii) After the treatment, operation or procedure, were you told the actual results of the treatment, operation or procedure in a way you could understand?
Condense 1.2	(i) Did a member of staff tell you about medication side effects to watch for when you went home?
	(ii) Were you told in a clear and understandable way how to take your medication?
**(2) Condense for Role-related Items**	
Condense 2.1	(i) When you had important questions to ask a doctor, did your doctor provide a clear and understandable answer to you?
	(ii) When you had important questions to ask a nurse, did the nurse provide a clear and understandable answer to you?
Condense 2.2	(i) Did you have confidence and trust in the doctors treating you?
	(ii) Did you have confidence and trust in the nurses treating you?
Condense 2.3	(i) How would you rate the care you received from the doctors?
	(ii) How would you rate the care you received from the nurses?
	(iii) How would you rate the care you received from the healthcare assistants?

### Stage 2: Validation Survey

Seven-hundred and thirty-five patients were approached and 516 patients completed the survey, giving a 70.2% response rate. The demographic characteristics of the respondents are given in Table 3. Their mean age was 63 years (S.D: 17.5 years, range 18–99 years) and half of them (50%) were men. Compared with the corresponding discharge population of 22,019 cases with a mean age of 65 years during the study period, the respondents were significantly younger (p<0.05) and fewer lived in old age homes (p<0.05). In terms of education level, 50.4% had a primary education or below, 39.0% had a secondary education and 10.3% had a post-secondary/tertiary education or above. The length of stay in hospital was 1 week or less for 77.7% of the patients and more than 1 week for 22.3%.

**Table 3:** Demographic characteristics of respondents and target population.

Demographics^a^		No. (%) of patients		
		(unless otherwise indicated)		
		Respondents	Target discharge population^b^	P value^c^
		(n = 516)	(n = 22,019)	
**Gender**	Male	258 (50.0)	11,284 (51.3)	0.575
**Age**	Mean ± standard deviation	62.9 ± 17.5	65.3 ± 18.3	<0.005
**Living in old-age home**	Yes	16 (3.1)	2,746 (12.5)	<0.001
**Education level**	No formal education or kindergarten	86 (16.7)	NA	NA
	Primary	174 (33.7)		
	Secondary (F.1-F.5)	201 (39.0)		
	Matriculation (F.6-F.7)	9 (1.7)		
	Post-secondary	8 (1.6)		
	Tertiary or above	36 (7.0)		
**Marital status**	Single	58 (11.2)	NA	NA
	Married	419 (81.2)		
	Divorced / Separated	26 (5.0)		
	Widow	12 (2.3)		
**Monthly household income**	<$5000	139 (26.9)	NA	NA
	$5000 to $9999	58 (11.2)		
	$10 000 to $14 999	36 (7.0)		
	$15 000 to $19 999	24 (4.7)		
	>$20 000	127 (24.6)		
	Not willing to answer / Don’t know	132 (25.6)		
**Working status^d^**	Retired	293 (56.8)	NA	NA
	Unemployed	35 (6.8)		
	Full-time student	4 (0.8)		
	Home-maker	42 (8.1)		
	Full-time worker / Part-time worker	134 (26.0)		
**Receiving any government allowance^e^**	Yes	266 (51.6)	NA	NA
**General health condition in past 4 weeks**	Very good	12 (2.3)	NA	NA
	Good	130 (25.2)		
	Fair	291 (56.4)		
	Poor	77 (14.9)		
	Very Poor	6 (1.2)		

^a^ Only three items of demographic characteristic (gender, age, and whether living in old-age home) could be retrieved from Hospital Authority dataset for the comparison between respondents and target discharged population; others were provided by the participants only

^b^ The target discharge populations were screened by Hospital Authority using the inclusion and exclusion criteria in the study; NA denotes not available

^c^ *t* test was carried to continuous variable such as age and Chi squared tests were carried to other categorical variables; NA denotes not available

^d^ These items do not add up to total 5030 due to missing data

^e^ Types of the government allowance included (1) Comprehensive Social Security Assistant, (2) disability allowance and (3) old-age allowance

#### Acceptability and Appropriateness

On average, the participants spent 18 minutes (S.D.: 4 minutes) and 9 minutes (S.D.: 2 minutes) completing the HKIEQ and short-form, respectively. The number of missing values ranged from 0.2% to 10% in the HKIEQ, whereas it was significantly reduced in the short-form, ranging from 0.2% to 1%.

#### Psychometric Properties: Construct Validity and Internal Reliability

To test the short-form’s construct validity, a Spearman’s rank correlation coefficient for the relationship between the summative scores in the short-form and the HKIEQ was calculated. The result was ρ = 0.92, which was statistically significant (p<0.05) and generally satisfactory, as recommended (ρ≥0.9) [11]. To test the internal reliability, the KR-20 coefficient of the summative scores across items within the core set was calculated: it was 0.86, which was higher than the recommended α≥0.7 [23,24]. The relationship between each individual item and the summative score of the short-form (item-to-total correlation, corrected with overlap) ranged from a Spearman correlation coefficient of 0.11 to 0.59; 17 out of 19 items (89%) complied with the recommended Spearman correlation coefficient (ρ) ≥0.3 [11,25]. The two items that fell below this recommended level were Item 1 Did you get the help you needed from staff? (eating meals, going to the toilet, movement in/out of bed), which achieved a correlation of 0.11, and Item 2 Did hospital staff tell you who to contact if you were worried about your condition or treatment after you left hospital?, which achieved a correlation of 0.23. When these two items were removed from the core set, the re-calculated Spearman’s rank correlation coefficient for the summative scores was slightly reduced from ρ = 0.92 to ρ = 0.90, which was still statistically significant (p<0.05), and the internal reliability improved a bit to α = from 0.70 to α = 0.87.

The survey responses to the condensed item Were you told in a clear and understandable way how to take your medication and about the side effects to watch for when you went home? indicated that around 33% of patients reported a problem with this experience. In the original HKIEQ, 13% and 38% of patients reported problems with taking medicine correctly and lacking information about side effects, respectively. To have a more precise and differential measures of patients’ experience, it was suggested that the condensed item be changed to match the two original items in the HKIEQ.

The final core set contained 18 items derived from the original HKIEQ, which mainly covered relational aspects of care along four dimensions of a patient’s journey: hospital staff, patient care and treatment, information on leaving hospital and overall impression (Table 4).

**Table 4:** Short-form Hong Kong Inpatient Experience Questionnaire.

Item	Item Content	Item total correlations
	**I Hospital Staff**	
1	Receiving a clear answer of the questions from doctor	0.4869
2	Having confidence/ trust in the doctors	0.5472
3	Receiving a clear answer of the questions from nurse	0.4646
4	Having confidence/ trust in nurses	0.5805
	**II Patient Care & Treatment**	
5	Receiving any information about the condition, treatment, operation or procedure and its results	0.5724
6	Having enough opportunity to talk to doctor by family member	0.3940
7	Discussing / comforting patient’s worries/fears by healthcare staff	0.4745
8	Any privacy when discussing condition/ treatment/procedure	0.4086
9	Doing everything to control pain by hospital staff	0.3056
	**III Information on Leaving Hospital**	
10	Telling how to take your medication	0.4800
11	Telling about medication side effects by hospital staff	0.4800
12	Telling danger signals watch for after discharge	0.5855
13	Giving family all information they needed for patient’s care and recovery	0.4349
	**IV Overall Impression**	
14	Being treated with respect and dignity in hospital	0.5149
15	Rating of the care received from doctors	0.5448
16	Rating of the care received from nurses	0.5333
17	Rating of the care received from healthcare assistants	0.4615
18	Rating of the overall care received	0.5823

### Discussion

This is the first study to validate a core set of measures for patient experience and satisfaction in Hong Kong. The core set of 18 items, named the Short-Form of the Hong Kong Inpatient Experience Questionnaire (SF-HKIEQ), was derived from the HKIEQ. Through rigorous research, we demonstrated that the SF-HKIEQ is a reliable and valid measure of key aspects of inpatients’ experience; although abbreviated from a much larger questionnaire, it maintains internal consistency and reliability.

That the short-form dramatically increased the survey’s acceptability was demonstrated by its maximum missing values of 1%, compared with the maximum missing values of 10% in the HKIEQ [14]. The SF-HKIEQ’s high reliability was evidenced by a Cronbach’s α (0.86) which gives it the same internal consistency as the original PPE-15 in England (0.86), and in various other countries (ranged from 0.80–0.87) [11,12]. The item-to-total correlations were also good; the recommended level of 0.3, corrected for overlap, was achieved for all of the items in the HKIEQ. The original version of the HKIEQ had a Spearman’s rank correlation coefficient of p = 0.75 [14]; the SF-HKIEQ had an even higher test-retest reliability in the 2-week interval of p = 0.92. The analysis demonstrated that the SF-HKIEQ is superior to HKIEQ for individual comparisons, implying its stability in repeated assessments.

Interestingly, the set of core items reflected HK patients’ concerns about the relational aspects of their care, such as privacy, respect, dignity and trust; receipt of clear and understandable information; availability of healthcare staff; emotional support and pain control; and information about medication side effects, danger signs to watch out for, and contact points for post-discharge support. These findings are similar to those of other short-form inpatient experience tools in Europe and the US [11,20,31,32]. In addition, the SF-HKIEQ further highlights patient concerns about post-discharge support, while the 15-item Picker Patient Experience Questionnaire (PPE-15) in the UK includes items on conflicting information given by healthcare staff and the extent of shared-decision making [11]. The 24-item Quality from the Patient’s Perspective (short-form of QPP) used in Sweden includes items on waiting time and room characteristics [31]. The 10-item Generic Short Patient Experiences Questionnaire (GS-PEQ) used in Norway includes items about waiting time, shared-decision making and safe care [32]; and the 18-item Short-Form Patient Satisfaction Questionnaire (PSQ-18) used in the US includes items on waiting time, safe care, right to access own medical records and having a business-like relationship with healthcare staff [20]. These differences in health systems, society, culture and values, and patients’ expectations about healthcare quality may influence the psychometric structure of the measuring tool. Our study, therefore, provides some new and important information.

Other aspects of patients’ experience of hospital care, such as access, food and cleanliness of the environment, which are not included in the SF-HKIEQ, reflected their lower priority for HK patients. Surprisingly, the issue of waiting time, which is highlighted in media headlines, was not regarded as a top priority by HK patients. In line with focus group findings, HK patients tend to be more accepting of the need to wait, indicating their appreciation of staff workloads and the pressures on the capacity of public healthcare provision [13].

The study has some limitations. First, the participants who were recruited for the validation survey were significantly younger and less likely to live in an old age home than the general discharge population. The results when this self-report measure is used to survey all of the users of a public hospital may be different. Second, although the SF-HKIEQ was proved to have good reliability and validity, it only captures the key aspects of inpatient experience, whereas the original version of the HKIEQ collects a comprehensive view of inpatient experience. However, the SF-HKIEQ’s has higher acceptability and may be more accurate than the HKIEQ when used for individual comparisons. Finally, our study used a cross-sectional design; longitudinal studies are needed to establish its sensitivity to change.

### Conclusion

The SF-HKIEQ, a locally validated core item scale, has been found to provide a good representative picture of key inpatient experiences in a hospital setting. It also provides an easy and quick mechanism for capturing hospitalization experiences that can be used for continuous quality improvement. The items in the questionnaire have a high degree of face validity, construct validity and internal reliability consistency. We suggest that the short-form instrument can be used to involve both healthcare staff and patients in the monitoring of the quality of hospital care and to heighten their awareness of this important aspect of patients’ lives.
